# Role of socioeconomic factors in developing mycetoma: Results from a household survey in Sennar State, Sudan

**DOI:** 10.1371/journal.pntd.0010817

**Published:** 2022-10-17

**Authors:** Natalia Hounsome, Rowa Hassan, Sahar Mubarak Bakhiet, Kebede Deribe, Stephen Bremner, Ahmed Hassan Fahal, Melanie J. Newport

**Affiliations:** 1 Brighton and Sussex Centre for Global Health Research, Brighton and Sussex Medical School, University of Sussex, Brighton, United Kingdom; 2 The Mycetoma Research Centre, University of Khartoum, Khartoum, Sudan; 3 Children’s Investment Fund Foundation, Addis Ababa, Ethiopia; 4 School of Public Health, College of Health Sciences, Addis Ababa University, Addis Ababa, Ethiopia; National Institute for Communicable Diseases, Johannesburg, South Africa, SOUTH AFRICA

## Abstract

**Background:**

Mycetoma is a chronic, progressively destructive disease of subcutaneous tissues and bones caused by certain species of bacteria or fungi. We conducted a cross-sectional community-based study alongside mapping of mycetoma in five administrative units with high mycetoma endemicity in the Eastern Sennar Locality, Sennar State, Sudan.

**Methods:**

A household survey was administered which included questions about the household members, household characteristics, economic activity and history of mycetoma. A clinical examination was conducted on all members of the household. If mycetoma was suspected, an individual questionnaire was completed collecting demographic, clinical and epidemiological data as well as information on the use of health care and associated costs. Geographical coordinates and photos of the lesions were taken, and the affected persons were referred to the medical centre for confirmation of the diagnosis and treatment. We compared the characteristics of households with confirmed cases of mycetoma with those without confirmed cases, and individuals with confirmed mycetoma with those in whom mycetoma was not confirmed.

**Results:**

In total 7,798 households in 60 villages were surveyed; 515 suspected cases were identified and 359 cases of mycetoma were confirmed. Approximately 15% of households with mycetoma had more than one household member affected by this disease. Households with mycetoma were worse off with respect to water supply, toilet facilities, electricity and electrical appliances compared to the survey households. Only 23% of study participants with mycetoma had sought professional help. Of these, 77% of patients travelled an average of six hours to visit a medical facility. More than half of patients had to pay towards their treatment. The estimated average cost of treatment was 26,957 Sudanese pounds per year (566 US dollars, exchange rate 2018).

**Conclusions:**

Results of this survey suggest that agricultural practices and reduced access to sanitation and clean water can be risk factors in developing mycetoma. Poor access to health care and substantial financial costs were barriers to seeking treatment for mycetoma.

## Author summary

Mycetoma is a neglected tropical disease caused by certain species of bacteria or fungi. The pathogens enter the body through a break in the skin and infect underlying soft tissues, leading to tissue destruction, loss of function, amputations and disability. Early diagnosis of mycetoma requires a clear understanding of disease prevalence, distribution and the contributing risk factors. In this study, we describe the results from a cross-sectional community-based study conducted in five administrative units with high mycetoma endemicity in the Eastern Sennar Locality, Sennar State, Sudan. 7,798 households in 60 villages were randomly selected across the entire locality. A household survey was administered and household members underwent clinical examination. In case of suspected mycetoma, geographical coordinates and photos of the lesions were taken, further demographic and clinical data were collected as was information on the use of health care and associated costs, and the individual was referred to the medical centre for confirmation of the diagnosis and treatment. This study aimed to identify socioeconomic risk factors contributing to mycetoma. We compared the characteristics of the households with confirmed cases of mycetoma with those without cases and individuals with confirmed mycetoma with those in whom mycetoma was not confirmed.

Our results suggest that agricultural practices and reduced access to clean water and sanitation may be risk factors in developing mycetoma. Poor access to health care and substantial financial costs may be barriers to seeking treatment for mycetoma.

## Introduction

Mycetoma is a neglected tropical disease caused by certain species of bacteria and fungi. It is endemic in many tropical and subtropical countries such as Sudan, Somalia, Senegal, India, Yemen, Mexico, Venezuela, Columbia and Argentina [1,2]. The pathogens enter the body through a break in the skin and infect underlying soft tissues, muscles and bones leading to tissue destruction, deformity, loss of function, amputation, disability and occasionally death. The disease is mainly painless in the early stages, therefore many patients present late to health care facilities with advanced infection. The foot and hand are the most frequently affected body parts, accounting for 82% of cases [3]. Socioeconomic factors are thought to play an important role in developing mycetoma. Mycetoma occurs more frequently in young working-age adult men (20–40 years) who practice agriculture barefooted [4]. The pathogens causing mycetoma are present in soil and water [5,6]. Cattle dung has also been suggested as a source of mycetoma pathogens [7]. The infection may result from inoculation via small injuries and thorn pricks [4,6]. In Sudan, animal enclosures made of thorny branches are frequently used to provide a safe place for livestock [8]. Areas harbouring *Acacia mellifera (*currently *Senegalia mellifera)* have been shown to overlap with the spatial distribution of mycetoma [9]. Populations living in endemic areas are advised to wear protective shoes, clear thorny bushes and keep animals in special enclosures [5,8].

Although the epidemiology of mycetoma is not fully understood, the disease can be effectively managed if diagnosed early. The diagnosis of mycetoma is based on clinical presentation, imaging studies and identification of the causative organisms in relevant clinical samples taken from affected tissues using fine-needle aspiration, or surgical biopsy. Microscopy and cytological, histopathological, immunohistochemical and molecular techniques based on the polymerase chain reaction (PCR) are applied to these samples [10]. Imaging including X-ray, ultrasound, MRI and CT scan examinations may be required to characterise the spread and extent of disease. These costly diagnostic modalities are only available in specialised medical centres located in big cities, which are not easily accessible to rural populations for a variety of reasons including the lack of infrastructure (e.g. roads that are unpassable during the rainy season) and cost of transport. Personal costs associated with the diagnosis and treatment of mycetoma are largely unknown. Religious beliefs and the use of traditional medicine by people with mycetoma are additional barriers to timely access to medical care [11].

Early diagnosis of mycetoma requires a clear understanding of disease prevalence, distribution and the contributing risk factors. A spatial geographical distribution study that mapped all cases of mycetoma presented to the Mycetoma Research Centre in Khartoum from the Eastern Sennar Locality, Sennar State, Sudan between 1991 and 2001 showed that most cases were located in the southern part of the locality along the Blue Nile river valley and its tributaries [12]. A modelling study of the spatial distribution of mycetoma among patients attending the Mycetoma Research Centre (University of Khartoum) between 1991 and 2018 suggested that aridity, proximity to water, low soil calcium and sodium levels, and the distribution of various species of thorny trees are strong predictors of the occurrence of mycetoma [13].

Here we report the results from the cross-sectional population survey conducted alongside spatial mapping across all five highly endemic administrative units of Eastern Sennar Locality, Sennar State, Sudan in 2019 [14]. The study estimated the overall prevalence of mycetoma to be 0.87% with cases clustered within the central and north-eastern part of the locality. The prevalence was highest in the 31–54 years old, and illiteracy, being married and having a history of skin trauma and thorn pricks were associated with mycetoma [14].

This study aimed to identify the socioeconomic factors contributing to mycetoma. Our objectives were: i) to compare characteristics of households with cases of mycetoma with those without cases, and ii) to compare individuals with confirmed mycetoma with those in whom mycetoma was not confirmed.

## Methods

### Ethics statement

Ethics approval for this study was obtained from the Mycetoma Research Centre, Khartoum, Sudan Institutional Review Board (Approval no. SUH 11/12/2018) and from the Brighton and Sussex Medical School Research Governance and Ethics Committee (ER/BSMS435/1). Written informed consent was obtained from each adult patient and parents or guardians of the population under 18 years old. Confirmed mycetoma cases were referred for management at Wad Onsa Regional Mycetoma Centre or the Mycetoma Research Centre (MRC).

### Study characteristics

**Fig 1:**
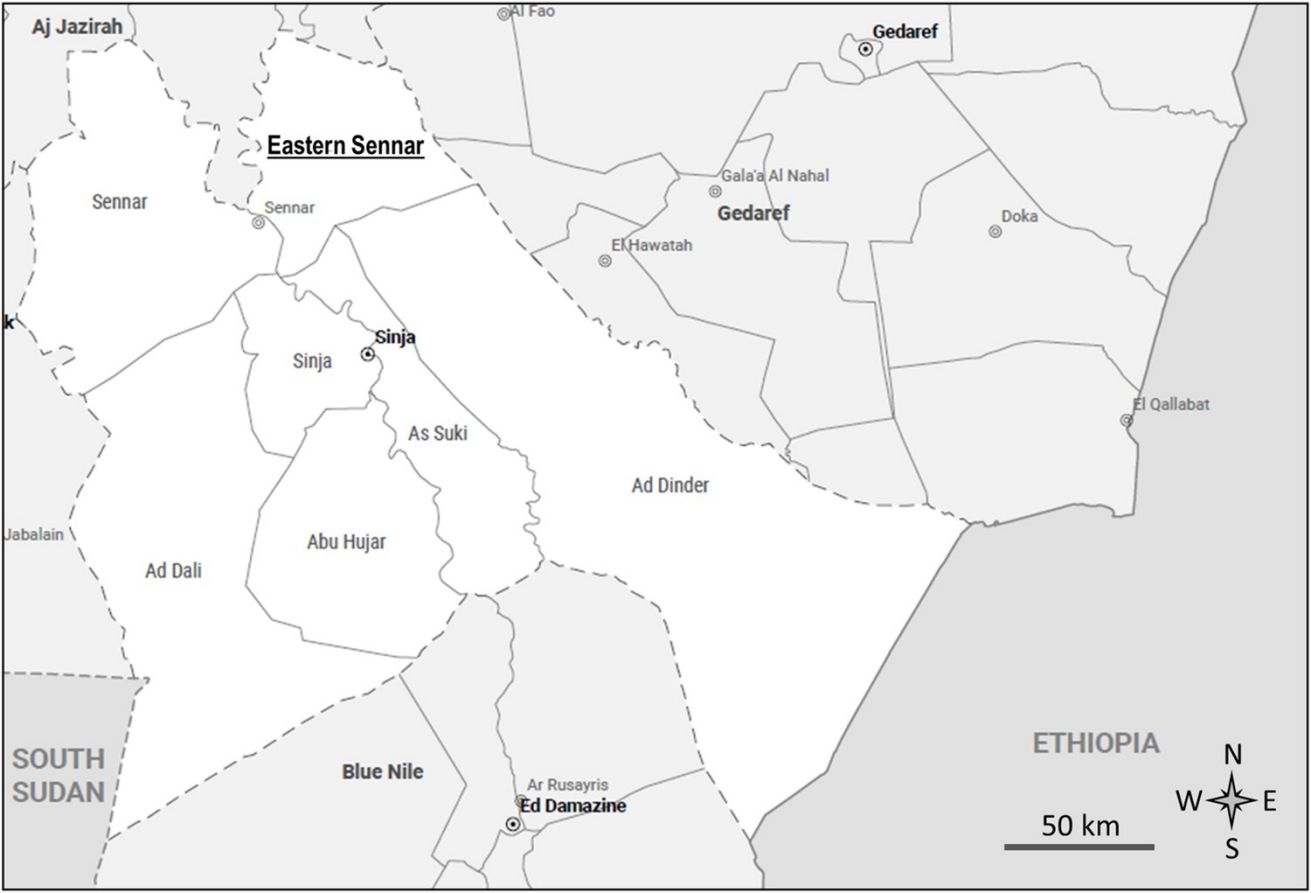
Administrative map of Sennar State, Sudan [15].

A detailed description of the survey is published elsewhere [14]. Briefly, a cross-sectional community-based survey was conducted in June-July 2019 in the Eastern Sennar locality, Sennar State, Sudan (Fig 1). The research area covered five administrative units with high mycetoma endemicity: Doba, El-Reif El-Shargi, Wad al Abbas, Wad Onsa and Wad Taktok. Sixty villages were randomly selected across the entire locality to participate in the survey. The sample size was calculated based on an estimated prevalence of 5 cases of mycetoma per 10,000 population, an average population size of the villages of 935 people (average household size of 7), the population of Eastern Sennar locality of 353,196 people, design effect of 2.5, the precision of 3 per 10,000 population and a community participation rate of 80%. To obtain a 95% confidence interval with a half-width of 3 per 10,000 population around an estimated prevalence of 5 per 10,000 requires 21,332 participants (53,330 upon inflating by the design effect of 2.5). Inflating this further for the anticipated 80% participation rate gives a total sample size of 66,663. Applying the finite population correction factor, nN/(n+N-1) (where N is population size 353,196 and n is the sample size 66,663) this becomes 56,079 which is rounded up to 56,100 i.e. 60 villages of 935 people.

### Survey population

The survey population included mainly subsistence farmers and their family members. All household members for whom informed consent was obtained were included in the survey. Written informed consent was obtained from the head of the household and verbal consent was obtained from each individual before the examination. There were no restrictions concerning age, sex, or health status.

### Survey process

A survey coordinator was appointed in each administrative unit who was responsible for the overall leadership, management, liaison and compliance of the project. A field logistics coordinator was responsible for moving the survey team (which included a coordinator, a doctor, and a medical assistant) between the villages, transporting suspected cases to Wad Onsa Mycetoma Centre for the confirmation of diagnosis, and providing study supplies. The coordinators were responsible for locating the targeted villages, making pre-survey arrangements, sensitizing regional leaders, explaining the objectives of the survey, community sensitization and mobilization. The data collectors were health assistants and doctors in the area who were responsible for case detection, data collection, and referral to the Wad Onsa Regional Mycetoma Centre. They were trained on field procedures, use of tablets, data entry and identification of mycetoma lesions. The coordinators visited the targeted villages before the actual day of the survey to accomplish the following tasks: to inform local leaders and community representatives about the objectives of the survey and to get permission, and to select a suitable day for the survey in consultation with the community members. A community member from each village was selected to assist the survey.

### Data collection

On the day of the survey, the team members visited the targeted household and provided a brief introduction to the head of the household and the household members about the purpose of the study. When one of the household members was missing during the survey administration the household was visited again up to three times. Written consent was taken from the head of the household. For illiterate individuals, a fingerprint was obtained. The data collectors started with the household survey including questions about the household members, household characteristics and economic activities (see the section below for more details). They also asked questions about the history of skin trauma and family history of mycetoma. After individual verbal consent was given, a clinical examination was conducted on all members of the household. For cases of suspected mycetoma (those with swelling of any part of the body or sinus formation with or without grain discharge), an individual questionnaire was completed asking about the disease symptoms, contacts with the environment and actions taken toward managing the disease. Geographical coordinates, as well as photos of the lesions, were collected. The individuals who were suspected to have mycetoma were transported to the regional Wad Onsa Mycetoma Cente by the study team for the confirmation of the diagnosis and treatment. They were provided a barcoded form which was scanned to link the questionnaire data with the results of subsequent investigations. The diagnosis was based on a combination of clinical and ultrasound examinations. Patients with grains, capsules, and the accompanying inflammatory granuloma on ultrasound scans in addition to clinical presentation (swelling and sinus formation with or without grain discharge) were classified as individuals with “confirmed mycetoma”. Those who presented with swelling or sinus formation but had negative ultrasound examinations were classified as individuals with non-confirmed mycetoma. Those in whom mycetoma diagnosis was not confirmed were referred for treatment to the nearest medical facility.

### Questionnaires

The household questionnaire included the personal details of the head of the household, the number of household members; the number of rooms in the household; types of materials used for the floor, walls and roof; type of cooking fuel used; toilet facilities; waste management; availability of electricity, drinking water and household appliances; vehicle ownership; land ownership, agricultural activities including growing crops, animal farming including types of animals, and animal shed location. Each household was assigned a unique identification number that could be linked to the individual questionnaires completed for all household members with suspected mycetoma.

The individual questionnaires collected information on age, place of birth; animals owned by the household; cultivated plants; species of trees in the vicinity of the household; the season of acquiring the disease, its duration and symptoms; the presence of other mycetoma cases in the household, contacts with health care professionals and associated costs; medication; hospitalisations; contact with traditional healers; money borrowed; the number of days unable to work because of the disease; and the number of days when help with household chores was required. The questionnaires were administered in Arabic.

All questionnaires were completed using tablet computers, checked for completeness and validity, and then data were transferred to the main server. Each questionnaire had a unique automatically generated identification number which was linked to the barcode on the referral form to the Wad Onsa Mycetoma Center.

### Data analysis

Data analysis was conducted in SPSS 25 (SPSS, Chicago, IL, USA) and Stata 17 (StataCorp, 2021). The questionnaire data were anonymised, checked for misspellings, and coded if required. The data from the individual questionnaire were merged with the data from the household questionnaire using the household identification number to enable comparisons of households with confirmed cases of mycetoma with households without confirmed cases. At the point of data analysis, the mycetoma diagnosis was not confirmed for 23 survey participants (21 of whom were traveling outside Sudan and two others who died). Therefore, the following analytical approaches were used:

1. The primary comparisons were conducted between the households with cases of mycetoma with those without cases, and individuals with confirmed mycetoma with those in whom mycetoma was not confirmed.
2. Additional analyses were conducted using imputed datasets to compare individuals with confirmed and non-confirmed mycetoma. Multiple imputations of missing diagnoses were conducted for the dataset including 23 participants who were lost to follow-up. Missing diagnoses were assumed to be missing at random. Five datasets were imputed for further analysis. Covariates included in the imputations were: the presence of swelling, sinuses and/or discharge, history of skin trauma, family history of mycetoma, and practices such as arable farming and animal grazing.

The statistical significance of differences between the samples was tested using mixed-effects models to adjust for the clustering effect. The household-level data were analysed using the “administrative unit” variable as a fixed effect and “village” as a random effect. The individual-level data were analysed using variables “village” and “household” as first- and second-level random effects. The models were compared using the likelihood-ratio test in Stata 17. Multivariable analysis was used for the multiple-choice questions.

Opportunity costs were calculated using the minimum monthly wage of 425 Sudanese pounds (SDG) [16] and 246 working days (2018). Costs were converted to US dollars (USD) using the exchange rate of 1 USD/47.65 SDG (December 31, 2018) [17].

## Results

### Socioeconomic characteristics of survey households

The data on household characteristics were collected from 7,798 households in 60 villages. A summary of the household characteristics is provided in Table 1. The number of people in a household varied from 1 to 35 with an average number of 5.6. The number of rooms in the household varied from 0 to 13. Most of the dwellings (79.0%) had 1–2 rooms.

**Table 1:** Socioeconomic characteristics of survey households (n = 7,798).

	Characteristics	n (%) unless indicated
**1**	**Number of surveyed households,** n = 7,798	
	Doba	1,397 (17.9)
	El-Reif El-Shargi	1,183 (15.2)
	Wad al-Abbas	463 (5.9)
	Wad Onsa	1,809 (23.2)
	Wad Taktok	2,946 (37.8)
**2**	**Number of people in household**, n = 7,798 mean (SD); median; min-max	5.6 (2.7); 5; 1–35
	1–3	1,784 (22.9)
	4–6	3,332 (42.7)
	7–10	2,375 (30.5)
	11 and more	307 (3.9)
**3**	**Number of rooms in household,** n = 7,797 mean (SD); median; min-max	1.9 (1.1); 2; 0–13
	0–2	6,159 (79.0)
	3–4	1,430 (18.3)
	5 and more	208 (2.7)
**4**	**Wall materials,** n = n = 7,798	
	brick	2,664 (34.2)
	concrete	212 (2.7)
	mud/dung	3,306 (42.4)
	stone	57 (0.7)
	wood	885 (11.3)
	no walls	2,122 (27.2)
**5**	**Floor materials,** n = 7,798	
	brick	30 (0.4)
	ceramic	174 (2.2)
	earth/sand	7,537 (96.6)
	other (e.g. plastic cover)	455 (5.8)
**6**	**Roof materials,** n = 7,798	
	cardboard	12 (0.2)
	concrete	2,045 (26.2)
	plastic	108 (1.4)
	traditional	6,331 (81.2)
	wood	467 (6.0)
	zinc	743 (9.5)
**7**	**Cooking fuel,** n = 7,798	
	animal dung	169 (2.2)
	coal	3,144 (40.3)
	gas	3,234 (41.5)
	*electricity*	34 (0.4)
	wood/plants	6,093 (78.1)
	Balanites aegyptiaca*	159 (2.6)
	Capparis decidua*	157 (2.6)
	*Eichhornia azurea*	74 (1.2)
	Faidherbia albida*	7 (0.1)
	*Gossypium barbadense*	1,419 (23.3)
	*Henlianthus annuus* (sunflower)	419 (6.9)
	*Indigofera oblongifolia*	410 (6.7)
	*Lens culinaris* (lentil)	87 (1.4)
	Prosopis chilensis*	242 (4.0)
	*Sorghum bicolor* (sorghum)	2,981 (48.9)
	Vachellia nilotica*	3,872 (63.5)
	Vachellia nubica*	684 (11.2)
	Vachellia seyal var. fistula*	1,161 (19.1)
	Vachellia seyal var. seyal*	1,134 (18.6)
	Ziziphus spina-christi*	704 (11.6)
**8**	**Water supply,** n = 7,798	
	piped	4,862 (62.3)
	tank	1,803 (23.1)
	well	1,905 (24.4)
	other (e.g. canal, river)	198 (2.5)
**9**	**Water purification,** n = 600	
	adding bentonite clay	396 (66.0)
	settling	192 (32.0)
	straining	11 (1.8)
	other (boiling, adding chemicals)	10 (1.7)
**10**	**Toilet facility,** n = 7,798	
	flush toilet	55 (0.7)
	pit latrine	3,328 (42.7)
	ventilated pit latrine	267 (3.4)
	no facility	4,141 (53.1)
**11**	**Waste disposal,** n = 7,798	
	burning	2,247 (28.8)
	burying	26 (0.3)
	throwing in a designated place	6,898 (88.5)
**12**	**Electricity,** n = 7,798	5,394 (69.2)
**13**	**Mobile phone,** n = 7,798	5,934 (76.1)
**14**	**Radio,** n = 7,798	1,711 (21.9)
**15**	**Refrigerator,** n = 7,798	2,162 (27.7)
**16**	**Television,** n = 7,798	3,419 (43.8)
**17**	**Transport,** n = 7,798	
	animal-drawn cart	1,965 (25.2)
	car or truck	844 (10.8)
	raksha	106 (1.4)
	No transport	5.218 (66.9)
**18**	**Agricultural land ownership,** n = 7,798	4,561 (58.5)
	Doba, n = 1,397	1,056 (75.6)
	El-Reif El-Shargi, n = 1,183	550 (46.5)
	Wad al-Abbas, n = 463	233 (50.3)
	Wad Onsa, n = 1,809	1,089 (60.2)
	Wad Taktok, n = 2,946	1,633 (55.4)
**19**	**Animal ownership**, n = 7,798	3,766 (48.4)
	Doba, n = 1,397	809 (57.9)
	El-Reif El-Shargi, n = 1,183	596 (50.4)
	Wad al-Abbas, n = 463	195 (42.1)
	Wad Onsa, n = 1,809	1,001 (55.3)
	Wad Taktok, n = 2,946	1,165 (39.5)
**20**	**Farm animals,** n = 3,776	
	camels	31 (0.8)
	chicken	835 (22.1)
	cows	985 (26.1)
	donkeys	870 (23.0)
	ducks	7 (0.2)
	goats	2,838 (75.2)
	horses	7 (0.2)
	pigeons	88 (2.3)
	rabbits	4 (0.1)
**21**	**Other animals,** n = 239	
	cats	20 (8.4)
	dogs	219 (91.6)
**22**	**Animal shed ownership,** n = 7,798	3,193 (40.9)
**23**	**Animal shed location,** n = 3,193	
	shed inside house	2,853 (89.4)
	shed outside house	828 (25.9)
	near house	471 (14.8)
	away from house	357 (11.2)

*thorny plant species. The percentages were rounded to one decimal place. Questions 4–11, 17, 20–22 and 23 were multiple-choice questions; the sum of percentages may exceed 100%.

### Dwellings

The walls of the dwellings were made of various materials including mud and animal dung (42.4%), brick (34.2%) and wood (11.3%). A small proportion of dwellings had concrete (2.7%) or stone walls (0.7%). Approximately 27% of dwellings had no built walls (semi-permanent structures). Nearly all (96.6%) of dwellings had earth or sand floors in at least a part of the house. Other materials such as bricks, ceramic tiles and plastic covers were occasionally used as flooring materials. The majority of roofs (81.2%) were of traditional style made of sticks, thatch and mud. Other roof materials included concrete (26.2%), zinc (9.5%), plastic (1.4%) and cardboard (0.2%).

### Cooking fuel

Wood/plants were used as cooking fuel in 78.1% of households; 41.5% of households used gas, 40.3% coal, 2.2% animal dung and 0.4% electricity. More than half of households (58%) relied on more than one source of cooking fuel. Wood was the sole source of cooking fuel in 40% of households.

### Water supply

Piped water (from a central pipe in the village) was available in 62.3% of households, well water in 24.4%, tank water in 23.1% of households and 2.5% of households used river water. Approximately 36% of households used water from multiple sources. Only 7.7% of households purified water for drinking. The most common purification methods were adding bentonite clay to absorb impurities [18] and settling. Straining, boiling and adding chemical agents (chlorine and alum) were rarely used (<1%).

### Sanitation

Approximately half of the surveyed households (53.1%) had no toilet facilities; 42.7% had pit latrines, 3.4% ventilated pit latrines, and 0.7% had flush toilets.

The main approach to waste disposal was throwing in a designated place (88.5%), followed by burning (28.8%) and burying (0.3%). Approximately 13% of households combined different ways of waste disposal.

### Electricity and household appliances

Electricity was available in 69.2% of households, television in 43.8%, radio in 21.9%; refrigerator in 27.7% and mobile phone in 76.1% of households.

### Transport

Vehicles were owned by 33.1% of households. These were animal-drawn carts (25.2%), cars/trucks (10.8%) and “rakshas”—three-wheeled motorised rickshaw-type vehicles (1.4%).

### Agricultural activities

Overall, approximately 58.5% of households owned agricultural land, varying from 46.5% in El-Reif El-Shargi to 75.6% in Doba. Farm animals were raised by 48.4% of households, from 39.5% in Wad Taktok to 57.9% in Doba. The commonest farm animals owned were goats, cows, donkeys, chickens, pigeons, camels, horses, rabbits and ducks. Approximately 41% of households had animal sheds. The majority of sheds were inside houses (89.4%). Outside sheds were located near the houses (14.8%) or further away from the houses within the boundaries of the village (11.2%) (Table 1).

### Comparison of households with cases of mycetoma with households without cases

Data for 7,206 households were available for comparative analysis (identification numbers allowing to link household-level data to individual-level data were missing for 592 households). Out of 7,206 households, 399 households with suspected mycetoma were identified, and for 288 households the mycetoma diagnosis was confirmed. Out of these, 234 households had one mycetoma case per household; 38 households had two cases; 10 households had three cases; four households had four cases, and two households had six cases. Table 2 shows a comparison between the households with confirmed cases of mycetoma and households without confirmed cases. The proportion of households with mycetoma varied among the survey areas from 2.3% in Wad Taktok to 11.9% in Wad al-Abbas. There were no significant differences between the households with confirmed cases of mycetoma and households without confirmed cases with respect to the number of people in the household, or the number of rooms for living. The proportion of households with piped and well water was lower in the mycetoma group (Table 2). Households with mycetoma were more likely to use tank water. Toilet facilities were unavailable in 66.3% of households with cases of mycetoma compared to 55.0% in households without cases. There were some differences in waste disposal between the two groups with burning and burying used more often in households with cases of mycetoma. The latter households were less likely to have electricity and electrical appliances (radio, refrigerator and television) compared to the households without mycetoma cases, although these differences were not statistically significant. Ownership of agricultural land, farm animals and animal sheds did not differ significantly between the two groups (Table 2).

**Table 2:** Comparison of households with confirmed cases of mycetoma and households without confirmed cases.

Characteristics	Households with mycetoma cases (n = 288)	Households without mycetoma cases (n = 6,918)	*p*-value
**Number of surveyed households,** n (%)			N/A
Doba	55 (4.4)	1,200 (95.6)	
El-Reif El-Shargi	31 (3.5)	850 (96.5)	
Wad al Abbas	55 (11.9)	408 (88.1)	
Wad Onsa	82 (4.7)	1,660 (95.3)	
Wad Taktok	65 (2.3)	2,800 (97.7)	
**Number of people in household** mean (SD); median; min-max	5.4 (2.4); 5; 1–14	5.5 (2.7); 5; 1–35	0.18
**Number of rooms in household** mean (SD); median; min-max	1.8 (1.0); 2; 1–6	1.9 (1.1); 2; 0–13	0.12
**Water supply**, n (%)			0.02
piped	159 (55.2)	4,236 (61.2)	
tank	102 (35.4)	1,685 (24.4)	
well	44 (15.3)	1,766 (25.5)	
other (e.g. river, canal)	2 (0.7)	212 (3.1)	
**Toilet facility**, n (%)			<0.01
flush toilet	1 (0.3)	47 (0.7)	
pit latrine	85 (29.5)	2,820 (40.8)	
ventilated pit latrine	11 (3.8)	236 (3.4)	
no facility	191 (66.3)	3,808 (55.0)	
**Waste disposal**, n (%)			0.03
burning	82 (28.5)	1,910 (27.6)	
burying	2 (0.7)	19 (0.3)	
throwing in a designated place	240 (83.3)	6,145 (88.8)	
**Cooking fuel**, n (%)			0.18
animal dung	8 (2.8)	160 (2.3)	
coal	96 (33.3)	2,788 (40.3)	
gas	118 (41.0)	2,784 (40.2)	
wood/plants	223 (77.4)	5,567 (80.5)	
other (e.g. electricity)	2 (0.7)	16 (0.2)	
**Electricity**, n (%)	184 (63.9)	4,788 (69.2)	0.19
**Mobile phone**, n (%)	224 (77.8)	5,220 (75.5)	0.17
**Radio**, n (%)	49 (17.0)	1,441 (20.8)	0.12
**Refrigerator**, n (%)	65 (22.6)	1,871 (27.0)	0.16
**Television**, n (%)	107 (37.2)	2,968 (42.9)	0.18
**Transport**, n (%)			
animal-drawn cart	90 (31.3)	1,759 (25.4)	
car or truck	32 (11.1)	730 (10.6)	
raksha	4 (1.4)	97 (1.4)	
no transport	178 (61.8)	4,634 (67.0)	
**Agricultural land ownership**, n (%)	164 (56.9)	3,999 (57.8)	0.16
**Farm animal ownership,** n (%)	143 (49.7)	3,304 (47.8)	0.19
**Farm animals**, n (%)			
camels	0 (0.0)	31 (0.4)	
chicken	27 (9.4)	725 (10.5)	
cows	43 (14.9)	833 (12.0)	
donkeys	34 (11.8)	816 (11.8)	
goats	107 (37.2)	2,520 (36.4)	
pigeons	2 (0.7)	75 (1.1)	
other (ducks, horses, rabbits)	0 (0.0)	17 (0.2)	
**Animal shed ownership**, n (%)	121 (42.0)	3.193 (41.0)	0.19
**Animal shed location**, n (%)			0.18
inside house	98 (34.0)	2,236 (32.3)	
outside house	42 (14.6)	693 (10.0)	
near house	27 (9.4)	411 (5.9)	
away from house	15 (5.2)	275 (3.7)	

The percentages were rounded to one decimal place. For the multiple-choice questions, the sum of percentages may exceed 100%. *p*-values were derived using mixed-effects models with an administrative unit as a fixed effect and a village as a random effect. Multiple-choice questions were analysed using multivariable analysis.

### Health care costs of patients with mycetoma

Table 3 summarises the use of health care services and associated costs, money borrowing and daily activities by people with confirmed mycetoma over the past year. The majority of survey participants with mycetoma (76.9%) did not seek professional help with respect to their disease. Out of 83 patients who contacted health care professionals, 75 had an appointment with a doctor, 7 with a medical assistant, and one patient was seen by a nurse. The average cost of contacts with health care professionals was 7,271 SDG (153 USD) per person per year. Approximately 77.1% of these patients (64/83) had to travel outside their village to see a health care specialist. On average, they spent six hours traveling to a medical facility. The majority of these patients (82.8%, 53/64) had to pay for their travel. A third of these patients were accompanied by another person. Approximately 5.8% of patients with mycetoma had been hospitalised in the previous year, and more than half of them had additional expenses such as food or overnight stay for the accompanying person. The average out-of-pocket expenses associated with hospitalisation were 8,182 SDG (172 USD) per person.

**Table 3:** Use of healthcare services and associated costs, money borrowing and daily activities over the past year by people with confirmed mycetoma (n = 359).

Activity	Number of users n (%)	Paid for services n (%)	Cost, SDG mean (min-max),
**Contacts with healthcare specialists**	83 (23.1)	60 (16.7)	7,271 (5–100,000)
doctor	75 (20.9		
medical assistant	7 (1.9)		
nurse	1 (0.3)		
no contact	276 (76.9)	N/A	N/A
**Travel to healthcare specialists**	64 (17.8)	53 (14.8)	1,086 (2–10,000)
**Hospital admissions**	21 (5.8)	18 (5.0)	8,182 (250–29,000)
**Medication**	14 (3.9)	14 (3.9)	9,369 (60–50,000)
**Contacts with traditional healers**	38 (10.6)	25 (7.0)	1,048 (10–5,000)
**Borrowed money**	28 (7.8)	N/A	4,814 (250–30,000)
**Daily activities**			
number of people with reduced activities	53 (14.8)	N/A	N/A
number of people required help	37 (10.3)	N/A	N/A
number of people helped by children	19 (5.3)	N/A	N/A
number of days completely unable to work, mean (min-max)	73 (1–365)	N/A	1,513 (21–5,100)
number of days required help, mean (min-max)	65 (1–365)	N/A	1,348 (21–5,100)

Costs are rounded to the nearest Sudanese pound (SDG)

Only 3.9% of people with mycetoma received medication with an average cost of 9,369 SDG (197 USD) per year. Traditional healers were attended by 10.6% of patients, with an average cost of 1,048 SDG (22 USD) per year. Approximately 7.8% of families had to borrow money from relatives or the community to meet their needs. The average amount of borrowings was 4,814 SDG (101 USD) per year. The estimated average cost of treatment was 26,975 SDG (567 USD) per year.

Approximately 15% of survey participants with mycetoma reported a reduction in daily activities due to their disease. They were completely unable to work on average for 73 days a year. The opportunity cost of illness estimated using the minimum monthly wage of 425 SDG [16] was 1,513 SDG (32 USD) per year. Approximately 10.3% of patients required help with everyday chores on average 65 days per year with an opportunity cost of 1,348 SDG (28 USD). Approximately 5.3% of patients were helped by children. (Table 3)

### Comparison of individuals with confirmed mycetoma with individuals in whom mycetoma was not confirmed

Out of 515 survey participants with suspected mycetoma, 359 had their diagnosis confirmed. Mycetoma was excluded in 133 participants (other diagnoses in this group included foreign body granuloma, fibroma and lipoma). Twenty-three participants did not attend for further investigation to confirm the presence or absence of mycetoma. The proportion of people with confirmed mycetoma varied between the administrative units from 2.8% in El-Reif El-Shargi to 37.6% in Wad Taktok. Table 4 summarises the characteristics of participants with confirmed and non-confirmed mycetoma. The average age of people with confirmed mycetoma was 30.0 (median 27) years, compared to 27.8 (median 25) years in those without mycetoma. The proportion of people with a family history of mycetoma was higher in the confirmed group (35.7%) than in the non-confirmed group (29.5%). The two groups were similar with respect to the number of household members (on average 5 people) and the number of rooms (on average 2 rooms). There were no statistically significant differences between the groups with regard to water supply, toilet facilities, waste disposal, utilities and appliances, vehicles and farm ownership.

**Table 4:** Comparisons of individuals with confirmed and non-confirmed mycetoma.

Characteristics	Confirmed mycetoma (n = 359)	Non-confirmed mycetoma (n = 133)	*p*-value
**Number of individuals**, n (%)			N/A
Doba	80 (22.3)	57 (42.9)	
El-Reif El-Shargi	10 (2.8)	0 (0.0)	
Wad al Abbas	30 (8.4)	1 (0.8)	
Wad Onsa	104 (29.0)	47 (35.3)	
Wad Taktok	135 (37.6)	28 (21.2)	
**Age, years,** mean (SD); median; min-max	30.0 (16.3); 27; 1–85	27.5 (17.1); 25; 5–100	0.68
**Family history of mycetoma**, n (%)	128 (35.7)	38 (28.6)	0.31
**Number of people in household** mean (SD); median; min-max	5.6 (2.4); 6; 1–14	5.4 (2.5); 5; 1–13	0.58
**Number of rooms in household** mean (SD); median; min-max	1.9 (1.0); 2; 1–6	1.9 (1.2); 2; 1–9	0.49
**Water supply**, n (%)			0.06
piped	193 (53.8)	76 (57.1)	
tank	127 (35.4)	47 (35.3)	
well	54 (15.0)	24 (18.0)	
other (e.g. river, canal)	37 (10.3)	5 (3.8)	
**Toilet facility**, n (%)			0.14
flush toilet	1 (0.3)	0 (0.0)	
pit latrine	107 (29.8)	32 (24.1)	
ventilated pit latrine	13 (3.6)	10 (7.5)	
no facility	234 (65.2)	91 (68.4)	
**Waste disposal**, n (%)			0.22
burning/burying	104 (29.0)	38 (24.4)	
throwing in a designated place	295 (82.2)	123 (92.5)	
**Electricity**, n (%)	208 (57.9)	68 (51.1)	0.42
**Mobile phone**, n (%)	277 (77.2)	106 (79.7)	0.51
**Radio**, n (%)	63 (17.5)	39 (29.3)	0.04
**Refrigerator**, n (%)	73 (20.3)	32 (24.1)	0.30
**Television**, n (%)	125 (34.8)	49 (36.8)	0.34
**Transport**, n (%)			0.42
animal-drawn cart	116 (32.3)	32 (24.1)	
car or truck	43 (12.0)	18 (13.5)	
raksha	5 (1.4)	3 (2.3)	
no vehicles	218 (60.7)	91 (68.4)	
**Land ownership**, n (%)	240 (66.9)	80 (60.2)	0.60
**Practicing agriculture**, n (%)	233 (64.9)	72 (54.1)	0.03
**Regularly practicing agriculture**, n (%)	84 (23.4)	24 (18.0)	0.86
**Type of agriculture**, n (%)			0.69
manual	224 (62.4)	69 (51.9)	
mechanical	5 (1.4)	1 (0.8)	
**Years of practicing agriculture,** mean (SD); median; min-max	14.1 (14.1); 8.0; 0–65	12.3 (12.9); 5; 1–60	0.75
**Crops**, n (%)			0.62
cotton	53 (14.8)	14 (10.5)	
millet	27 (7.5)	1 (0.8)	
sorghum	165 (46.0)	58 (43.6)	
wheat	59 (16.4)	14 (10.5)	
other (e.g. sunflower, sesame and vegetables)	92 (25.6)	18 (13.5)	
**Practice animal grazing**, n (%)	106 (29.5)	46 (34.6)	0.30
**Animal ownership,** n (%)	182 (50.7)	56 (42.1)	0.13
**Farm animals**, n (%)			0.89
chicken	36 (10.0)	10 (7.5)	
cows	58 (16.2)	15 (11.3)	
donkeys	42 (11.7)	15 (11.3)	
goats	138 (38.4)	40 (30.1)	
pigeons	2 (0.6)	1 (0.8)	
sheep	34 (9.5)	8 (6.0)	
**Trees on the land**, n (%)	167 (46.5)	44 (33.1)	0.12
**Animal shed ownership**, n (%)	155 (43.2)	62 (46.6)	0.98
**Shed location**, n (%)			0.74
inside house	125 (34.8)	49 (36.8)	
outside house	53 (14.8)	21 (15.8)	
near house	34 (9.5)	13 (9.8)	
away from house	19 (5.3)	8 (6.0)	

The percentages were rounded to one decimal place. For the multiple-choice questions, the sum of percentages may exceed 100%. *p*-values were derived using mixed-effects models using variables “village” and “household” as first- and second-level random effects, respectively. For the multiple-choice questions, multivariable analysis was used.

More than half of the respondents in both groups were arable farmers. The most frequently cultivated crops were sorghum, wheat, cotton and millet. Other crops included sunflowers, sesame and various fruit and vegetables (e.g. tomatoes, cucumbers and watermelons). The proportion of people involved in agricultural activities was significantly higher in those with mycetoma (64.6%) compared to those who did not have mycetoma (54.9%). People with mycetoma were more likely to practice agriculture regularly (23.4% versus 17.3%), although this difference was not statistically significant.

Over a third of respondents were involved in raising animals, goats, cows, donkeys, chickens, sheep and pigeons. There were no statistically significant differences between the two groups regarding the types of animals raised, although the overall livestock ownership was higher in the confirmed mycetoma group (42.3% vs 36.6%). Animal sheds were owned by 43.2% of households with mycetoma cases and by 46.2% of households without mycetoma cases. No statistically significant differences were found with respect to the shed ownership and location (inside/outside or near/away from homes). The majority of animal sheds were located inside the houses (34.8% in the confirmed group and 35.9% in the group without mycetoma)

The proportion of land with trees was higher in the confirmed mycetoma group (46.5% vs 33.3%). Thorny trees comprised 90% of species growing on the land. The proportion of thorny tree species in the vicinity of the household was also higher in the confirmed mycetoma group (42.9% vs 28.2%) (Table 4).

### Comparison of imputed datasets for the groups with confirmed and non-confirmed mycetoma

Multiple imputations were conducted for 23 survey participants who were unavailable for the confirmation of the diagnosis. The resulted dataset included 373 confirmed diagnoses and 142 not confirmed diagnoses. S1 Appendix summarises the characteristics of the populations with and without a diagnosis of mycetoma. Comparisons of imputed data revealed similar results to the analysis of non-imputed datasets. People with mycetoma were more likely to practice agriculture, have mycetoma in their family, grow farm animals and use animal-drawn vehicles, although these differences did not reach statistical significance (S1 Appendix).

## Discussion

### Role of economic factors in developing mycetoma

The burden of mycetoma is heavily concentrated in low- and middle-income countries. Poverty is often seen as a root cause of neglected tropical diseases because of its association with deprived living and working conditions, and limited access to health care services. This survey was conducted in rural, remote and economically-deprived areas in Sudan. The surveyed households were lacking essential utilities such as electricity (30% of households), piped water (38%) and toilet facilities (53%). Less than half of households owned a TV (44%) and less than a third (28%) had a refrigerator. Animal-drawn carts were the dominant type of vehicle (67%). The average number of people in a household was 5.6 and the average number of rooms was 2. Drinking water was purified in less than 10% of households. Despite widespread overall poverty, the households with confirmed cases of mycetoma were worse off with respect to the supply of water and electricity, toilet facilities, and ownership of electrical appliances (Table 2)

In agreement with previous studies, this survey found that the prevalence of mycetoma was higher in the populations involved in agricultural activities (Table 4). Sennar state is located in central Sudan with an area of 40,680 square kilometers. Almost half of this territory is a low rainfall savannah with a total area of rainfed agriculture of approximately 23,000 square kilometers [19]. Both traditional rain-fed and modern irrigated farming are practiced in Sudan [20], but the majority of people affected by mycetoma come from rural, isolated communities involved in manual agriculture. They are more likely to contract injuries when working in the field without protective footwear. Our survey has demonstrated that 64.6% of people affected by mycetoma practice subsistence farming growing sorghum, millet, wheat, maize, barley and various vegetables, and raising cattle, camels, goats and sheep. Animal farming has previously been identifed as a contributing factor in developing mycetoma [4,7]. In Sudan, animals are often kept inside homes or in outside enclosures surrounded by thorny tree branches. The floors of the animal enclosure are covered with animal dung and thorns, which can cause skin injuries and inoculation with mycetoma pathogens [8]. It has been suggested that animal dung can promote the growth of environmental microorganisms that cause mycetoma [7]. Results of this survey suggest that families with mycetoma are more likely to own farm animals and animal sheds, although no statistically significant differences were found with respect to the animal ownership, type of farmed animals or animal shed location between populations with and without confirmed mycetoma cases (Table 4).

### Role of sanitation in developing mycetoma

The results of our survey suggest that poor sanitation and limited access to clean water are factors contributing to mycetoma. Lack of toilet facilities in economically deprived communities increases the risk of contracting waterborne infections such as cholera, dysentery, hepatitis A, typhoid and polio, as well as wound infections. According to the UNICEF report on water, sanitation and hygiene in Sudan [21], approximately 34.6% of households in Sudan practice open defecation. Our study has demonstrated that over half of households in the survey area (53.1%) lacked toilet facilities (Table 1). This proportion was higher (66.3%) in the households with confirmed mycetoma (Table 2.)

The shortage of clean drinking water in rural Sudan contributes to poor domestic hygiene. According to published data, 68% of households in Sudan have access to improved water sources such as piped water, protected wells and boreholes [21]. Coverage for piped water in the survey area was less than the national coverage (62.3% of households), and only 55.2% of households with cases of mycetoma had access to piped water (Table 2). Person-to-person and water-borne modes of pathogen transmission typically associated with poor sanitation have not been reported for mycetoma. However, poor sanitation and lack of clean water may facilitate the acquisition of mycetoma in individuals predisposed by skin trauma, which could explain multiple cases of mycetoma in some households, especially those which cannot afford footwear. A family history of mycetoma was reported in 13% of patients who underwent surgical treatment for mycetoma at the Mycetoma Research Centre, Khartoum, Sudan in the period 1991–2015 [22], and 15% of children seen at the Centre during 1991–2009 [23]. Results of our survey showed that 35.7% of people with confirmed mycetoma reported a family history of the disease (Table 4), and 18.8% of households with confirmed mycetoma had more than one affected household member. This could reflect the shared environment that families are exposed to but could also be explained by genetic factors harboured by relatives within the same household [22,24].

### Role of environmental factors

Mycetoma is endemic in many tropical and subtropical countries where the climate is characterised by low annual rainfall, a dry winter season, a relatively short but heavily rainy summer season, and high year-round temperatures. It is thought that these conditions may contribute to the survival of microorganisms that cause mycetoma [25]. Analyses of soil samples collected from highly endemic areas in Khartoum State, Sudan confirmed the presence of *Madurella mycetomatis* DNA fragments identical to those from biopsies of patients with mycetoma [6]. *Madurella mycetomatis* eumycetoma is the most common cause of mycetoma in Sudan, accounting for around 70% of cases [6]. The prevalence of these fungi in soil samples suggests that soil may be the prime reservoir from which the infection originates. Our survey shows that the majority of mycetoma infections (83%) were acquired during the rainy seasons (summer and autumn), which can be due to the high prevalence of mycetoma-causing pathogens in wet soil.

The abundance of thorny trees in endemic areas could contribute to skin injuries through which pathogens can penetrate [4,9]. Our study revealed that 70% of farms in endemic areas were surrounded by trees, of which 90% of species had thorns. These include a group of *Vachellia* species as well as other thorny plants such *as Balanites aegyptiaca*, *Capparis decidua*, *Prosopis chilensis* and *Ziziphus spina-christi*. Branches from thorny trees are used by the farmers to build animal enclosures to secure livestock [8]. In this survey, we found that trees were used as the main source of cooking fuel in more than 70% of households. Trees were also used as a building material, for example, in traditional roofs found in over 80% of households (Table 1). Activities such as working in the field without protective footwear, collecting fuel wood, repairing roofs and animal enclosures pose a significant risk of skin injuries allowing entry of pathogens that are present in the environment.

### Access to health care

Lack of qualified medical help and substantial healthcare costs are important factors contributing to the progression of mycetoma. Our survey showed that almost 80% of people with suspected mycetoma did not seek professional help. There are several reasons for this. Due to the lack of pain at the onset of mycetoma patients tend to present at later stages of the disease when surgical treatment (including amputation) may be required. The analysis of 6,792 patients with mycetoma managed at the Mycetoma Research Centre of the University of Khartoum, Sudan showed that over half of them (57%) had previous surgery elsewhere with limited success [22]. The identification of the mycetoma-specific pathogen is a prerequisite for the successful treatment of the disease (for example, differentiating bacterial and fungal causes which require different antimicrobial treatments). The methods allowing identification of mycetoma pathogens as well as techniques required to localise the main focus and extent of disease are not widely available in Sudan. Our study showed that patients with suspected mycetoma have to travel long distances to the nearest health care facility. Many patients need to be accompanied by another person. Over half of patients had to pay towards their treatment with cost being a further barrier. The average annual cost of treatment including consultations, hospitalisations, travel, medication, and help from traditional healers amounted to 26,957 SDG (566 USD) per year (Table 3). Such expenses are unaffordable for many households. For comparison, the minimum monthly wage in Sudan in 2018 was 425 SDG (9 USD) [16]. According to our survey, approximately a third of people who sought medical help with respect to suspected mycetoma had to borrow on average 4,249 SDG (90 USD) per year to meet their needs and the needs of their families.

### Comparison with published studies

The majority of epidemiological data on mycetoma come from case reports and single-centre studies. Large population studies are costly and labour-intensive, and therefore rare. A large historic survey of 2,150 cases (1956–1985) was carried out in Mexico to determine the incidence and epidemiological characteristics of the population affected by mycetoma [26]. This survey was updated to 3,933 cases in 2012 [27]. The collected data included age, sex, occupation, geographical area, affected body area and aetiological agent. The affected population were predominantly farmers and their family members, 76.1% male and 16–50 years old [26,27].

A retrospective epidemiological study of 264 cases (1981–2000) was carried out in West Bengal, India [28]. The population characteristics were similar to those reported in the previous studies and included agricultural workers, predominantly male (68.6%) with mycetoma onset between 16 and 25 years and a history of pricks and skin trauma. Actinomycetoma was the most common type of mycetoma in this population(74.6%) [28].

A retrospective review of biopsy reports for 279 mycetoma cases (1950–2019) was conducted to estimate mycetoma prevalence in Uganda [29]. A mapping study of 337 mycetoma cases (1993–2016) was conducted in Senegal [30]. However, no demographic or socioeconomic data were reported in these studies [29,30].

In Sudan, a study of the spatial geographical distribution of mycetoma was conducted using data from 594 patients with confirmed mycetoma (1991–2020) [12]. Demographic characteristics were collected alongside geographic information including geological, soil, temperature and land cover details. The main group affected by mycetoma included 20–40 years old males (77.9%), aged <40 years at presentation, with eumycetoma being the most common type of mycetoma (82%) [12].

The current study (see also the accompanying paper [14]) presents, to our knowledge, the most comprehensive characterisation of the population affected by mycetoma. In addition to clinical epidemiology data, we provided characteristics of households, economic activities and use of health care services by people with mycetoma. The results from our study suggest that socioeconomic factors in combination with the natural occurrence of mycetoma pathogens contribute to the high mycetoma prevalence in Sudan.

## Conclusions

Results of this survey suggest that agricultural practices and reduced access to sanitation and clean water can be risk factors in developing mycetoma. Poor access to health care and substantial financial costs were barriers to seeking treatment for mycetoma.

### Limitations

1. The diagnosis of mycetoma is based on clinical presentation, typical radiological findings and microbiological identification of the causative organisms (e.g. using microscopy and cytological, histopathological, immunohistochemical and molecular techniques) [10]. In the current study, the diagnosis was based on a combination of clinical and ultrasound examinations since mycetoma grains and the accompanying inflammatory granulomata have a characteristic ultrasonic appearance [31]. Given that microbiological identification of the mycetoma-causative pathogen was not possible due to the lack of available facilities locally, there was a possibility of not confirming true cases of mycetoma in individuals who presented with swelling or sinus formation but had negative ultrasound examinations.
2. At the time of data analysis, 23 out of 515 cases with suspected mycetoma were not confirmed due to the loss to follow-up. The primary analysis compared cases with a confirmed diagnosis of mycetoma to those with another diagnosis combined with those lost to follow-up. This means that there is a possibility of positive mycetoma cases among those lost to follow-up. To address this uncertainty, multiple imputations were conducted for participants with a missing diagnosis. Analysis using the imputed data (S1 Appendix) revealed similar results to the analysis of the non-imputed datasets (Table 4).
3. To identify factors associated with mycetoma we compared individuals with confirmed cases of mycetoma with those in whom mycetoma had been not confirmed and an alternative diagnosis was made. This group was not necessarily representative of the general study population and bias may have been introduced to the analysis. However, this group is a useful comparator as individual data for the general population was not collected in this survey.
4. Due to the multiple comparisons of survey variables (Tables 2 and 4), some statistically significant differences may be found by chance.

## Supporting information

S1 Appendix Comparison of imputed datasets for the populations with confirmed and non-confirmed mycetoma. (DOCX)
