# Supplementary material for: Role of socioeconomic factors in developing mycetoma: Results from a household survey in Sennar State, Sudan
Source: PLoS Negl Trop Dis. 2022 Oct 17;16(10):e0010817. doi: 10.1371/journal.pntd.0010817 (PMC9624402; doi:10.1371/journal.pntd.0010817)
Supplement: S1 Appendix — (DOCX) [file pntd.0010817.s001.docx]

**S1 Appendix.** Comparison of imputed datasets for the populations with confirmed and non-confirmed mycetoma

| **Characteristics** | **Positive mycetoma (n=373)** | **Negative mycetoma (n=142)** | ***p*-value** |
| --- | --- | --- | --- |
| **Number of individuals**, n (%) |  |  | N/A |
| Doba | 83 (22.3) | 62 (43.7) |  |
| El-Reif El-Shargi | 10 (2.7) | 0 (0.0) |  |
| Wad al Abbas | 30 (8.0) | 2 (1.4) |  |
| Wad Onsa | 108 (29.0) | 48 (33.8) |  |
| Wad Taktok | 142 (38.1) | 30 (21.1) |  |
| **Age, years,**  mean (SD); median; min-max | 29.8 (16.3); 27; 1-85 | 27.9 (17.3); 25; 5-100 | 0.79 |
| **Family history of mycetoma**, n (%) | 133 (35.7) | 41 (28.9)  ) | 0.26 |
| **Number of people in household**  mean (SD); median; min-max | 5.5 (2.4); 5; 1-14 | 5.4 (2.5); 5; 1-13 | 0.64 |
| **Number of rooms in household**  mean (SD); median; min-max | 1.9 (1.1); 2; 1-9 | 1.9 (1.2); 2; 1-6 | 0.96 |
| **Water supply**, n (%) |  |  | 0.09 |
| piped | 198 (53.1) | 80 (56.3) |  |
| tank | 131 (35.1) | 50 (35.2) |  |
| well | 57 (15.3) | 26 (18.3) |  |
| other (e.g. river, canal) | 39 (10.5) | 6 (4.2) |  |
| **Toilet facility**, n (%) |  |  | 0.69 |
| flush toilet | 1 (0.3) | 0 (0.0) |  |
| pit latrine | 114 (30.6) | 33 (23.2) |  |
| ventilated pit latrine | 13 (3.5) | 10 (7.0) |  |
| no facility | 240 (64.3) | 99 (69.7) |  |
| **Waste disposal**, n (%) |  |  | 0.21 |
| burning/burying | 108 (29.0) | 24 (23.9) |  |
| throwing in a designated place | 305 (81.8) | 131 (92.3) |  |
| **Utilities and appliances**, n (%) |  |  |  |
| electricity | 217 (58.2) | 71 (50.0) | 0.27 |
| mobile phone | 288 (77.2) | 113 (79.6) | 0.73 |
| radio | 64 (17.2) | 39 (27.5) | 0.09 |
| refrigerator | 77 (20.6) | 33 (23.2) | 0.45 |
| television | 131 (35.1) | 50 (35.2) | 0.65 |
| **Transport**, n (%) |  |  | 0.34 |
| animal-drawn cart | 123 (33.0) | 34 (23.9) |  |
| car or truck | 43 (11.5) | 19 (13.4) |  |
| raksha | 5 (1.3) | 3 (2.1) |  |
| no vehicles | 225 (60.3) | 97 (68.3) |  |
| **Land ownership**, n (%) | 248 (66.5) | 87 (61.3) | 0.27 |
| **Practicing agriculture**, n (%) | 241 (64.6) | 78 (54.9) | 0.04 |
| **Regularly practicing agriculture**, n (%) | 86 (23.1) | 25 (17.6) | 0.99 |
| **Type of agriculture**, n (%) |  |  | 0.18 |
| manual | 232 (62.2) | 74 (52.1) |  |
| mechanical | 5 (1.3) | 1 (0.7) |  |
| **Years of practicing agriculture,**  mean (SD); median; min-max | 14.1 (14.1); 9.0; 0-65 | 12.0 (12.5); 5.5; 1-60 | 0.61 |
| **Crops**, n (%) |  |  | 0.66 |
| cotton | 54 (14.5) | 14 (9.9) |  |
| millet | 27 (7.2) | 2 (1.4) |  |
| sorghum | 169 (45.3) | 64 (45.1) |  |
| wheat | 63 (16.9) | 14 (9.9) |  |
| other (e.g. sunflower, sesame and  vegetables) | 93 (24.9) | 19 (13.4) |  |
| **Practice animal grazing**, n (%) | 112 (30.0) | 53 (37.3) | 0.09 |
| **Animal ownership,** n (%) | 190 (50.9) | 57 (40.1) | 0.05 |
| **Farm animals**, n (%) |  |  | 0.95 |
| chicken | 37 (9.9) | 10 (7.0) |  |
| cows | 59 (15.8) | 16 (11.3) |  |
| donkeys | 44 (11.8) | 16 (11.3) |  |
| goats | 144 (38.6) | 41 (28.9) |  |
| pigeons | 2 (0.5) | 1 (0.7) |  |
| sheep | 35 (9.4) | 9 (6.3) |  |
| **Trees on the land**, n (%) | 170 (45.6) | 49 (34.5) | 0.23 |
| **Animal shed ownership**, n (%) | 163 (43.7) | 64 (41.0) | 0.80 |
| **Shed location**, n (%) |  |  | 0.70 |
| inside house | 131 (70.4) | 50 (69.4) |  |
| outside house | 55 (29.6) | 22 (30.6)  ) |  |
| near house | 36 (19.4) | 14 (19.0) |  |
| away from house | 19 (10.2) | 8 (11.1) |  |

The percentages were rounded to one decimal place. For the multiple-choice questions, the sum of percentages may exceed 100%. *p*-values were derived using a mixed-effects model using variables “village” and “household” as first- and second-level random effects, respectively. Responses to multiple-choice questions were analysed using multivariable analysis.
